# Path-Following Control of Wheeled Planetary Exploration Robots Moving on Deformable Rough Terrain

**DOI:** 10.1155/2014/793526

**Published:** 2014-03-26

**Authors:** Liang Ding, Hai-bo Gao, Zong-quan Deng, Zhijun Li, Ke-rui Xia, Guang-ren Duan

**Affiliations:** ^1^State Key Laboratory of Robotics and Systems, Harbin Institute of Technology, Harbin 150080, China; ^2^Key Lab of Autonomous System and Network Control, College of Automation Science and Engineering, South China University of Technology, Guangzhou 510640, China

## Abstract

The control of planetary rovers, which are high performance mobile robots that move on deformable rough terrain, is a challenging problem. Taking lateral skid into account, this paper presents a rough terrain model and nonholonomic kinematics model for planetary rovers. An approach is proposed in which the reference path is generated according to the planned path by combining look-ahead distance and path updating distance on the basis of the carrot following method. A path-following strategy for wheeled planetary exploration robots incorporating slip compensation is designed. Simulation results of a four-wheeled robot on deformable rough terrain verify that it can be controlled to follow a planned path with good precision, despite the fact that the wheels will obviously skid and slip.

## 1. Introduction

Wheeled mobile robots (WMRs) are typical nonholonomic systems and they have attracted the attention of many researchers as they do not satisfy Brockett's necessary condition [1]. A considerable amount of research related to the control of WMRs has been carried out, addressing issues such as posture stabilization, path following, and trajectory tracking, in which perfect constraints with no longitudinal or lateral wheel skid were usually assumed to exist [2–6].

Despite the rich results that have been obtained in studies of WMRs that applied ideal assumptions, new control problems arose with the development of WMRs for deployment in challenging terrain, such as the planetary exploration rovers. The Mars exploration rovers, Sojourner, Spirit, and Opportunity of the USA have greatly enhanced our knowledge horizon [7] and they have led to a surge in planetary exploration using WMRs. In the future, the Mars rovers of the MSL and ExoMars missions and the lunar rovers [8] of the SELENE and Chang'e missions will be launched. In contrast with the assumed properties of conventional terrain, that is, “hard” and “flat,” planetary terrain is deformable and rough [9]. As a result, the assumptions of “single-point contact” and “no longitudinal or lateral skid” are negated for the planetary WMRs. Actually, it is obvious that when moving on such a rough and deformable terrain the wheels will experience longitudinal slip and lateral skid, causing the rover to deviate from the planned path, lose efficiency, and even get stuck [9, 10].

**Figure 1:**
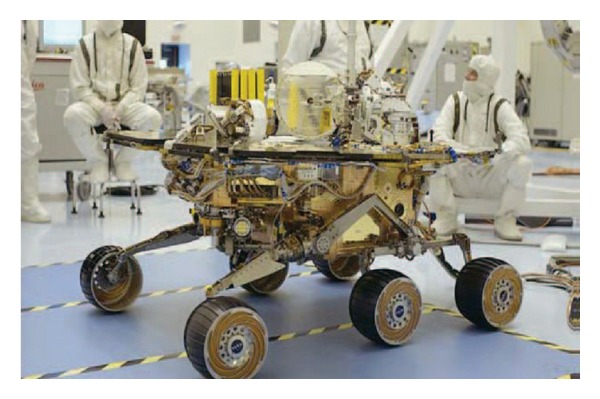
Flight rover Spirit [11].

In addition to the longitudinal slip and lateral skid of wheels moving on rough and deformable terrain, the redundant control of different wheels is another challenging problem. The current planetary exploration rovers have four or six independently driving wheels, and the four wheels at the corners are independently steering wheels, as shown in Figure 1 [11]. Iagnemma et al. presented a physics-based control strategy for planetary rovers, considering the kinematics, wheel-terrain interaction mechanics, dynamics (quasistatic), terrain geometry, and so on, in order to increase their tractive performance [12]. This control concept is in contrast to the conventional approach, which uses limited or no physical systems information. A rough terrain control (RTC) method is developed by exploiting the actuator redundancy of multiple wheels to improve the tractive performance and reduce power consumption [13]. A control algorithm the objective of which is to keep the slip ratios of the wheels within a small value and limit excessive wheel force was proposed by Yoshida and Hamano in order to increase the traversing ability of the rovers and avoid their digging into the soil or getting stuck [14]. An optimal torque control method is presented by Lamon et al. for the six-wheeled Shrimp rover based on the Hertz-Föppl model to calculate the resistance force of the soil [15, 16]. In order to save the energy and time expended by a planetary rover moving on deformable rough terrain, a control approach was developed in order to keep the slip ratios of all the wheels equal and the velocity of the body constant by compensating for the slip [17].

The above-mentioned research mainly concerns the redundant control of planetary rovers, with the objective of improving their traversing performance by coordinating the velocity or torques of the driving wheels, while the path-following problem is solved by coordinating the velocity or position of the different steering wheels. A steering maneuver strategy for a four-wheeled rover tested on lunar soil regolith simulant was investigated under different steering angles using both dynamics simulation and experiments [18]. A path-following algorithm that provides both steering and driving maneuvers was developed to direct a rover to follow a path by compensating for the lateral slip [19]. The rough terrain is simplified to the different slopes with which a rover has contact. However, the path-following problem of a planetary rover on very rough terrain has been little researched.

This study focuses on the path-following problem of a planetary rover on deformable and very rough terrain, that is, when the wheels and the vehicle body are not at the same slope and their local orientation coordinates differ. A nonholonomic kinematics model of planetary rovers traversing deformable rough terrain and a control strategy that coordinates the different steering wheels to realize path-following control of planetary rovers on challenging terrain are presented. The control algorithm is verified using a high-fidelity simulation platform [20].

## 2. Modeling of WMRs on Deformable Rough Terrain

### 2.1. Geometry Modeling of Wheel-Terrain Contact

For the sake of simplicity, studies in the literature often assume that wheel-terrain interaction occurs at a single point beneath the center of the wheel. This simplification will, however, lead to large errors when a WMR traverses over deformable rough terrain. On the one hand, the contact area between the wheel and the soil is large enough to need to be considered; on the other hand, it is determined by the geometry of the terrain rather than by the point beneath the wheel's center. The local coordinates of the contact areas and the wheels should be calculated, as they are indispensable for the kinematics modeling of WMRs on rough and deformable terrain. For instance, the direction of a wheel's velocity is approximately parallel to the contact surface rather than the horizontal plane.

**Figure 2:**
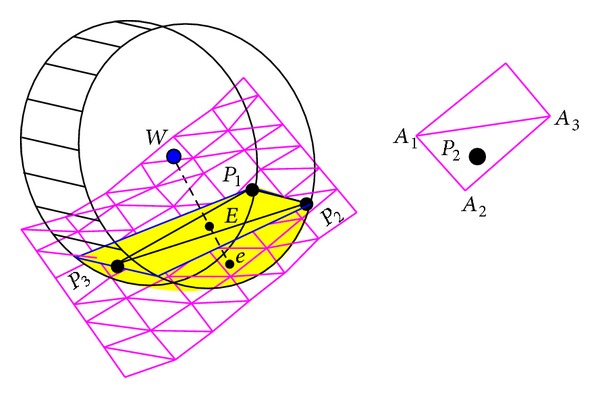
Contact area of a wheel moving on rough and deformable terrain [20].

Figure 2 shows the contact area of a wheel moving on rough and deformable terrain. The coordinate of a wheel's center *W* is denoted by (*x*_*w*_, *y*_*w*_, and *z*_*w*_). The terrain is characterized by a digital elevation map (DEM) so that the coordinates of all the mesh grids are known. The contact area can be simplified to an inclined plane, which is determined by three boundary points *P*_1_ (*x*_1_, *y*_1_, and *z*_1_), *P*_2_ (*x*_2_, *y*_2_, and *z*_2_), and *P*_3_ (*x*_3_, *y*_3_, and *z*_3_). The normal vector of plane *P*_1_ *P*_2_ *P*_3_ is

(1) $$\begin{array}{c} z_{e}=\left[\begin{array}{c} A_{t}\\ B_{t}\\ C_{t} \end{array}\right]=\left[\begin{array}{c} x_{2}-x_{1}\\ y_{2}-y_{1}\\ z_{2}-z_{1} \end{array}\right]\times \left[\begin{array}{c} x_{3}-x_{1}\\ y_{3}-y_{1}\\ z_{3}-z_{1} \end{array}\right]. \end{array}$$

The equation of the inclined plane *P*_1_ *P*_2_ *P*_3_ is therefore

(2) $$\begin{array}{c} A_{t}\left(x-x_{1}\right)+B_{t}\left(y-y_{1}\right)+C_{t}\left(z-z_{1}\right)=0. \end{array}$$

Line *WE* is perpendicular to the plane *P*_1_ *P*_2_ *P*_3_, where *e* is the end point at both line *WE* and the wheel's surface. The wheel sinkage *Ee* can be calculated using analytic geometry according to the coordinates of *W* and the equation of the inclined plane, *P*_1_ *P*_2_ *P*_3_ [20]. In terms of control, the local coordinates of the wheels are more important.

Figure 2 shows the coordinates and slope angles of a wheel moving on the inclined plane *P*_1_ *P*_2_ *P*_3_. {Σ_*w*_} is the local coordinate system of the wheel, and {Σ_*e*_} is the coordinate system with the same orientation as {Σ_*w*_}; however, their origins are different, being at the end point *e* and wheel center *W*, respectively. **x**_*e*_ is the longitudinal direction of a wheel; **y**_*e*_ is its lateral direction; **z**_*e*_ is the normal direction of the wheel-soil contact plane.

**z**_*e*_ is calculated using (1). **x**_*e*_ is the intersection line between the wheel-soil contact plane and the plane with an included angle of *φ*_*w*_ with the *x*_*I*_-axis, where *φ*_*w*_ is the yaw angle of a wheel, which is controlled by the steering motor of the wheel. It is deduced that [20]

(3) $$\begin{array}{c} x_{e}=\left\{C_{t},C_{t}\tan\varphi_{w},-A_{t}-B_{t}\tan\varphi_{w}\right\}. \end{array}$$

The vector direction of *y*_*e*_ is thus determined as follows:

(4) $$\begin{array}{c} y_{e}=z_{e}\times x_{e}=\left[\begin{array}{c} -A_{t}B_{t}-\left(B_{t}^{2}+C_{t}^{2}\right)\tan\varphi_{w}\\ C_{t}^{2}+A_{t}\left(A_{t}+B_{t}\tan\varphi_{w}\right)\\ A_{t}C_{t}\tan\varphi_{w}-B_{t}C_{t} \end{array}\right]. \end{array}$$

The transformation matrix from {Σ_*e*_} to {Σ_*I*_} is calculated with **x**_*e*_, **y**_*e*_, and **z**_*e*_:

(5) Aw=AwI=AeI=[CtX1−AtBt−(Bt2+Ct2)tanφwX2AtX3CttanφwX1Ct2+At2+AtBttanφwX2BtX3−At−BttanφwX1AtCttanφw−BtCtX2CtX3],

where

(6) X1=Ct2(1+tan2φw)+(At+Bttanφw)2X2=X3[At2+Ct2+2AtBttanφt+(Bt2+Ct2)tan2φw]X3=At2+Bt2+Ct2.

**Figure 3:**
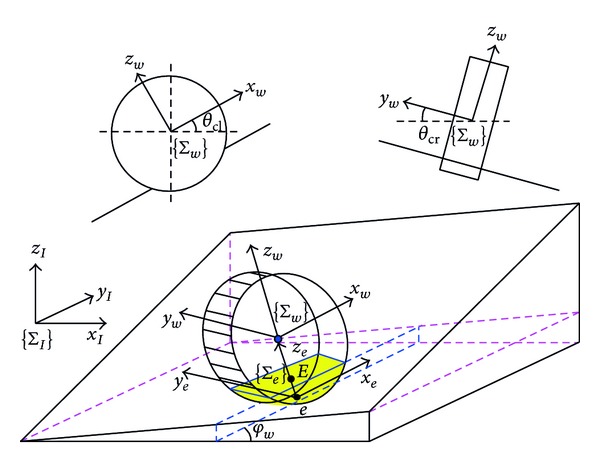
Coordinates and slope angles of a wheel moving on the inclined plane.

In Figure 3, *θ*_cl⁡_ and *θ*_cr_ denote the slope angles of a wheel climbing up and moving across, respectively. The roll, pitch, and yaw angles of a wheel on the inclined plane are then {*θ*_cr_, *θ*_cl⁡_, *φ*_*w*_}. *θ*_cl⁡_ and *θ*_cr_ can be calculated from

(7) $$\begin{array}{c} \theta_{\mathrm{cl}}=\arcsin\left[\frac{-A_{t}-B_{t}\tan\varphi_{w}}{X_{1}}\right]\\ \theta_{\text{cr}}=\arcsin\left[\frac{C_{t}\left(A_{t}\tan\varphi_{w}-B_{t}\right)}{X_{2}}\right]. \end{array}$$

When a virtual rover is being controlled in a numerical simulation, the coordinates of its wheel's center *W* and the angle of *φ*_*w*_ are known; the methods of calculating the coordinates of contact points *P*_1_, *P*_2_, and *P*_3_ are presented in [20]. The transformation matrix ^*I*^ **A**_*w*_ can be calculated using (5). When an actual rover is being controlled, the angles {*θ*_cr_, *θ*_cl⁡_, *φ*_*w*_} for all the wheels can be measured with sensors, and ^*I*^ **A**_*w*_ can also be calculated.

### 2.2. Nonholonomic Kinematics Model of WMRs on Deformable Rough Terrain

**Figure 4:**
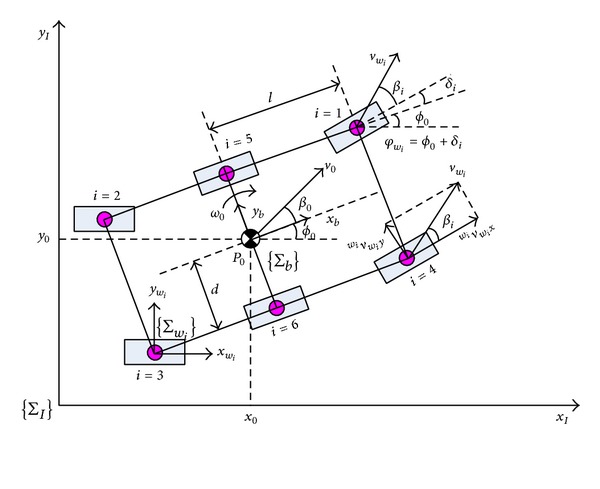
Model of a six-wheeled planetary rover.

As the terrain is rough and deformable, the wheels experience longitudinal slip and lateral skid and all the coordinates of the wheels and of the rover's vehicle are different in terms of not only position but also orientation. The nonholonomic kinematics model, which includes the properties of the terrain, vehicle, and wheels, constitutes the basis of path following. A model of a six-wheeled planetary rover is shown in Figure 4.

Lateral velocity that is perpendicular to the longitudinal direction of the vehicle body exists when a rover is moving on rough and deformable terrain. There is an included angle between the longitudinal velocity and the actual velocity of the vehicle, which is called the side skid angle and denoted by *β*_0_:

(8) β0=arctan⁡(y˙0bx˙0b).

Let *ϕ*_0_ denote the yaw angle of the vehicle's body. The nonholonomic kinematics model of the vehicle's body is

(9) $$\begin{array}{c} {\dot{y}}_{0}\cos\left(\phi_{0}+\beta_{0}\right)-{\dot{x}}_{0}\sin\left(\phi_{0}+\beta_{0}\right)=0. \end{array}$$

Let *β*_*i*_ denote the side skid angle of the *i*th wheel, that is, the included angle between the component velocity along the longitudinal direction of the wheel and its actual velocity. *δ*_*i*_ denotes the steering angle of the *i*th steering wheel; for a nonsteering angle, *δ*_*i*_ is always zero. Let *w*_*i*_ denote the local coordinate of the *i*th wheel; the nonholonomic kinematics equation of the wheel is then

(10) y˙wiwicos⁡βi−x˙wiwisinβi=0,

where x˙wiwi and y˙wiwi, respectively, denote the longitudinal and lateral velocity of the *i*th wheel in its local coordinate denoted by *w*_*i*_. These velocities can be determined by kinematics analysis of the planetary rover's movement on rough and deformable terrain.

**Figure 5:**
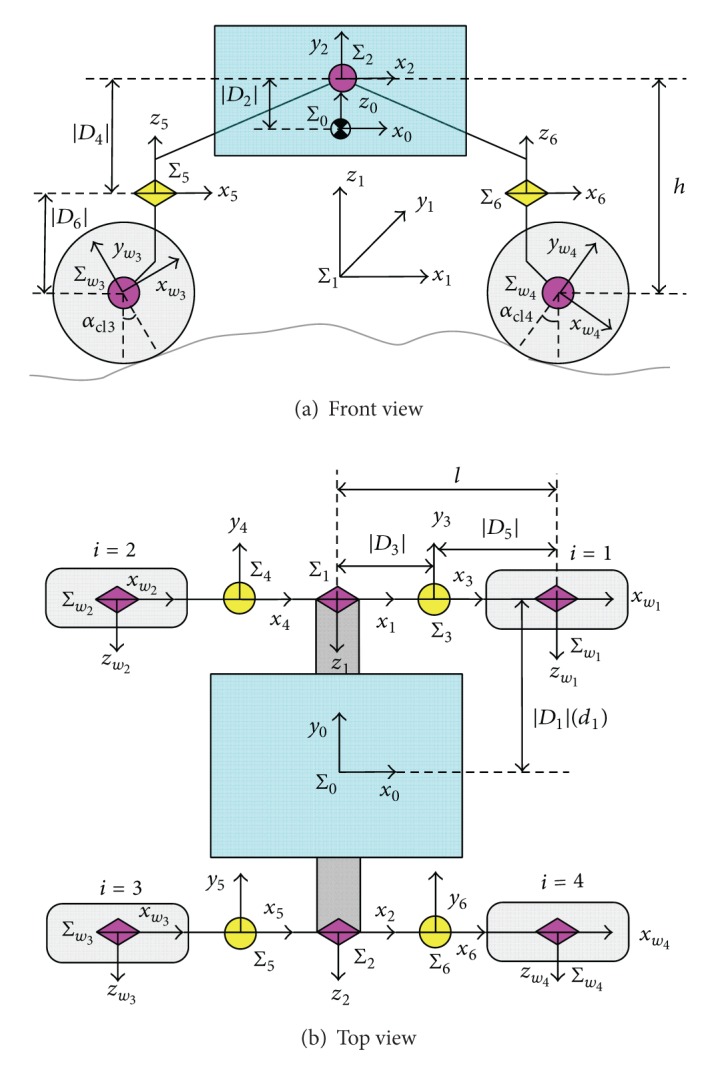
Coordinates of four-wheeled planetary rover.

In order to demonstrate the calculation of x˙wiwi and y˙wiwi, El-Dorado II, a four-wheeled planetary rover, the coordinates of which are shown in Figure 5, is taken as an example. On flat terrain, the orientations of coordinate systems Σ_1_ and Σ_2_ are the same as the orientation coordinate systems of Σ_3_, Σ_4_, Σ_5_, and Σ_6_ and those of Σ_*w*_1__(Σ_7_), Σ_*w*_2__(Σ_8_), Σ_*w*_3__(Σ_9_), and Σ_*w*_4__(Σ_10_). The routes from Σ_0_, the rover's center, to the wheels are Σ_0_ → Σ_1_ → Σ_3_ → Σ_*w*_1__, Σ_0_ → Σ_1_ → Σ_4_ → Σ_*w*_2__, Σ_0_ → Σ_2_ → Σ_5_ → Σ_*w*_3__, and Σ_0_ → Σ_2_ → Σ_6_ → Σ_*w*_4__. The model of calculating the velocity of Wheel 1 is deduced in detail.

According to [20], the velocity of Wheel 1 is

(11) vw1=v0+ω0×P0w1+A00A11Z1×P1w1q˙1 +A00A33Z3×P3w1q˙3,

where **v**_0_ and **ω**_0_ are the linear velocity and angular velocity of the vehicle body, respectively, *q*_*i*_ is the generalized coordinate of the *i*th joint, **P**_*ij*_ is the vector from joint *i* to joint *j*, and Zii=[001]T, denoting the projection coordinates on the *i*th coordinate system of the *z*-axis of joint *i*.

In (11), **A**_0_ is the orientation matrix of the rover's vehicle body and is the function of the RPY (roll, pitch, and yaw) angles (*ψ*_0_, *θ*_0_, *ϕ*_0_):

(12) $$\begin{array}{c} A_{0}=\left[\begin{array}{ccc} c\varphi_{0}c\theta_{0} & c\varphi_{0}s\theta_{0}s\psi_{0}-s\varphi_{0}c\psi_{0} & c\varphi_{0}s\theta_{0}c\psi_{0}+s\varphi_{0}s\psi_{0}\\ s\varphi_{0}c\theta_{0} & s\varphi_{0}s\theta_{0}s\psi_{0}+c\varphi_{0}c\psi_{0} & s\varphi_{0}s\theta_{0}c\psi_{0}-c\varphi_{0}s\psi_{0}\\ -s\theta_{0} & c\theta_{0}s\psi_{0} & c\theta_{0}c\psi_{0} \end{array}\right]. \end{array}$$

^0^ **A**_1_ and ^0^ **A**_3_ are the transformation matrixes from Σ_1_ to Σ_0_ and from Σ_3_ to Σ_0_, respectively:

(13) A10=[cq1−sq1000−1sq1cq10]A30=[cq1cq3−cq1sq3−sq1sq3cq30sq1cq3−sq1sq3cq1],

where *cq*_*i*_ = cos⁡(*q*_*i*_), *sq*_*i*_ = sin(*q*_*i*_), *q*_1_ and *q*_2_ are the joint angle coordinates of the suspension system, *q*_3_–*q*_6_ are the coordinates of the steering joints, and *q*_7_–*q*_10_ are the joint angle coordinates of the wheels.

**P**_0*w*_1__, **P**_1*w*_1__, and **P**_3*w*_1__ are the position vectors from the center of Wheel 1 to the origin of coordinates 0, 1, and 3, respectively. According to Figure 5, we obtain the following: P010=[0D1D2]T, P131=[D3D40]T, P373=[D50D6]T, P01=A0P001=A0[0D1D2]T, and

(14) P3w1=P37=A0A3  0P337=A0[D5c1c3−D6s1D5s3D5s1c3+D6c1]P13=A00A1P113=A0[D3c1−D4s10D3s1+D4c1]P1w1=P13+P3w1=A0[D3c1−D4s1+D5c1c3−D6s1D5s3D3s1+D4c1+D5s1c3+D6c1]P0w1=P01+P13+P3w1=A0[D3c1−D4s1+D5c1c3−D6s1D1+D5s3D2+D3s1+D4c1+D5s1c3+D6c1].

By substituting (12)–(14) into (11), the absolute velocity of Wheel 1 can be obtained. Using the corresponding parameters *D*_1_–*D*_6_ for all the wheels, the velocity of each wheel can also be obtained, using the same equation as for Wheel 1. For a typical four-wheeled rover with symmetric structure, such as the El-Dorado II rover of Tohoku University [21], only the length, width, and height of the rover are considered, which are denoted by 2*l*, 2*d*, and *h*, respectively. The corresponding *D*_1_–*D*_6_ are *D*_2_ = *D*_4_ = *D*_5_ = 0 and

(15) $$\begin{array}{c} D_{1}=\{\begin{array}{cc} d & \left(w1\right)\\ d & \left(w2\right)\\ -d & \left(w3\right)\\ -d & \left(w4\right), \end{array}\;\;D_{3}=\{\begin{array}{cc} l & \left(w1\right)\\ -l & \left(w2\right)\\ -l & \left(w3\right)\\ l & \left(w4\right), \end{array}\\ D_{6}=\{\begin{array}{cc} -h & \left(w1\right)\\ -h & \left(w2\right)\\ -h & \left(w3\right)\\ -h & \left(w4\right). \end{array} \end{array}$$

Let **v**_*w*_*i*_1_, **v**_*w*_*i*_2_, and **v**_*w*_*i*_3_ denote the velocity components caused by the angular velocity of the vehicle body, angular velocity of Joint 1, and angular velocity of Joint 3, respectively. They are expressed using (16)–(24):

(16) vwi1=ω0×P0wi=[0f1−f2−f10f3f2−f30][ψ˙0θ˙0ϕ˙0]

(17) vwi2=A00A11Z1×P1w1q˙1=q˙r[f4f5f6]T

(18) vwi3=A00A33Z3×P3w1q˙3=D6q˙3(A0[−s10c1]T)×(A0[−s10c1]T)=0.

*f*_1_ to *f*_6_ in (15) and (16) are

(19) f1=−sθ(D3cq1−D6sq1)+D1cθsψ +cθcψ(D3sq1+D6cq1)f2=sϕcθ(D3cq1−D6sq1)+D1(cϕcψ+sϕsθsψ) +(sϕsθcψ−cϕsψ)(D3sq1+D6cq1)f3=cϕcθ(D3cq1−D6sq1)+D1(cϕsθsψ−sϕcψ) +(cϕsθcψ+sϕsψ)(D3sq1+D6cq1)f4=(cϕsθcψ+sϕsψ)(D3cq1−D6sq1) −cϕcθ(D3sq1+D6cq1)f5=(sϕsθcψ−cϕsψ)(D3cq1−D6sq1) −sϕcθ(D3sq1+D6cq1)f6=cθcψ(D3cq1−D6sq1)+sθ(D3sq1+D6cq1).

Parameter ${\dot{q}}_{r}$ in (16) denotes the angular velocity of the suspension joints. For Wheels 1 and 2, ${\dot{q}}_{r}={\dot{q}}_{1}$, whereas, for Wheels 3 and 4, ${\dot{q}}_{r}={\dot{q}}_{2}$. The absolute velocity of the *i*th wheel is

(20) $$\begin{array}{c} v_{w_{i}}=\left[\begin{array}{c} {\dot{x}}_{w_{i}}\\ {\dot{y}}_{w_{i}}\\ {\dot{z}}_{w_{i}} \end{array}\right]=\left[\begin{array}{c} {\dot{x}}_{0}\\ {\dot{y}}_{0}\\ {\dot{z}}_{0} \end{array}\right]+\left[\begin{array}{ccc} 0 & f_{1} & -f_{2}\\ -f_{1} & 0 & f_{3}\\ f_{2} & -f_{3} & 0 \end{array}\right]\left[\begin{array}{c} \dot{\psi }\\ \dot{\theta }\\ \dot{\phi } \end{array}\right]+{\dot{q}}_{r}\left[\begin{array}{c} f_{4}\\ f_{5}\\ f_{6} \end{array}\right]. \end{array}$$

The velocity of the *i*th wheel in the coordination system of {Σ_*w*_*i*__} can be calculated using

(21) vwiwi=inv(Awi)vwi=AwiTvwi.

Substituting (5) into (21), one obtains the following:

(22) x˙wiwi=[x˙wiCtwi+y˙wiCtwitanφwi  +(−Atwi−Btwitanφwi)z˙wi]×(X1wi)−1y˙wiwi={x˙wi[−AtwiBtwi−(Btwi2+Ctwi2)tanφwi]  +y˙wi[Ctwi2+Atwi2+AtwiBtwitanφwi]  +z˙wi(AtwiCtwitanφwi−BtwiCtwi)}×(X2wi)−1z˙wiwi=x˙wiAtwi+y˙wiBtwi+z˙wiCtwiX3wi,

where *φ*_*wi*_ = *ϕ*_0_ + *δ*_*i*_, the yaw angle of the *i*th wheel, ${\left[\begin{array}{ccc} A_{twi} & B_{twi} & C_{twi} \end{array}\right]}^{T}$ is the normal vector of the slope that is in contact with the *i*th wheel, and *X*_1*wi*_, *X*_2*wi*_, and *X*_3*wi*_ are the functions of the normal vector calculated by (6).

By substituting (20) and (22) into (10), the nonholonomic kinematics equations of all the wheels are obtained.

## 3. Reference Path Generation Method

**Figure 6:**
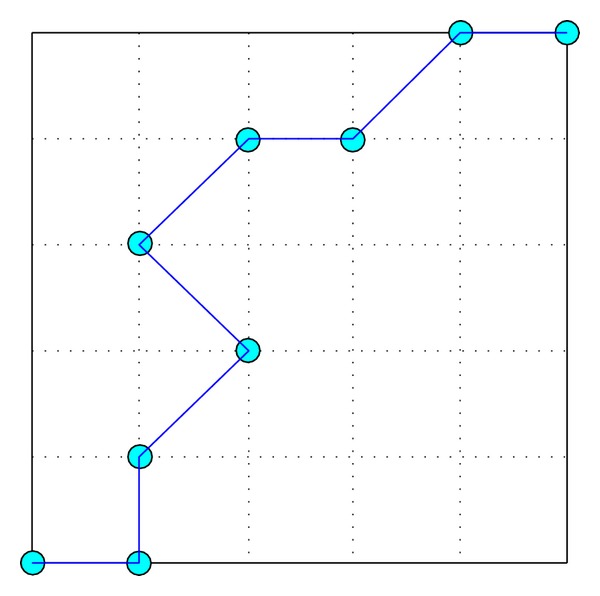
A planned path in DEM.

A rover's optimal path in challenging terrain can be planned on a digital elevation map (DEM) [22]. The coordinates of each point in the DEM are composed of three elements, *x*, *y*, and *z*. The *x* and *y* coordinates of each point on the path are planned; their *z* coordinates are determined by the terrain.  Figure 6 shows a typical planned path, which is characterized by coordinates in the horizontal plane and the yaw angle *ϕ*, that is, (*x*, *y*, *ϕ*).  Table 1 shows the position coordinates and yaw angles of the planned path delineated in Figure 6, where *x*_*r*_ and *y*_*r*_ are the resolutions of the DEM. The unit of *x*, *x*_*r*_, *y*, and *y*_*r*_ is meter (m), and the unit of *ϕ* is degree (°).

The rover's reference path is updated in real time based on the carrot following method according to the path planned in DEM. Let *R*_*f*_ denote the look-ahead distance of a planetary rover when moving forward. To describe circle, the center of the rover is used as the center and *R*_*f*_ as the radius; the forward intersection points of the circle with the planned path are then found. Next, the forward goal point toward which the rover should move from the intersection points is determined. The forward points are the points toward which the rover will move and do not include the points it has already passed. The following four types of situation should be considered when determining the goal points.

**Figure 7:**
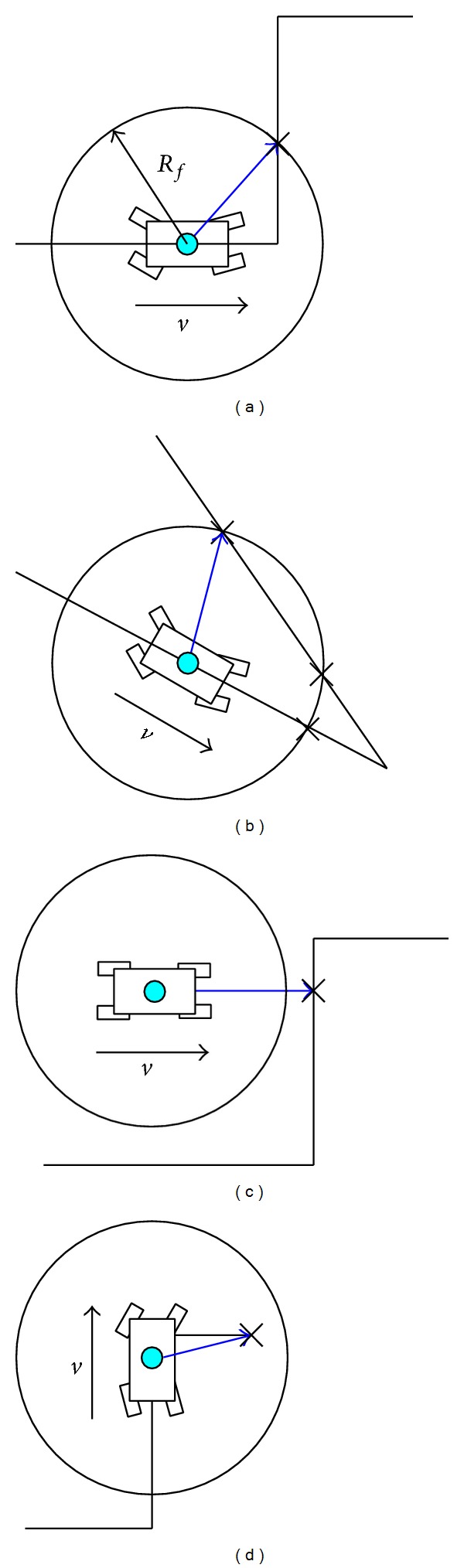
Classifications of goal points.

1. If there is only one forward intersection point, it is the goal point, as shown in Figure 7(a).
2. If there are several forward intersection points, the most forward one is the goal point, as shown in Figure 7(b).
3. If there is no forward intersection point and the shortest distance from the rover's center to the planned path is larger than *R*_*f*_, the forward point on the planned path that is the smallest distance from the rover's center is the goal point, as shown in Figure 7(c).
4. If there is no forward intersection point and the entire forward path is within the look-ahead circle, the destination point is the goal point, as shown in Figure 7(d).

**Figure 8:**
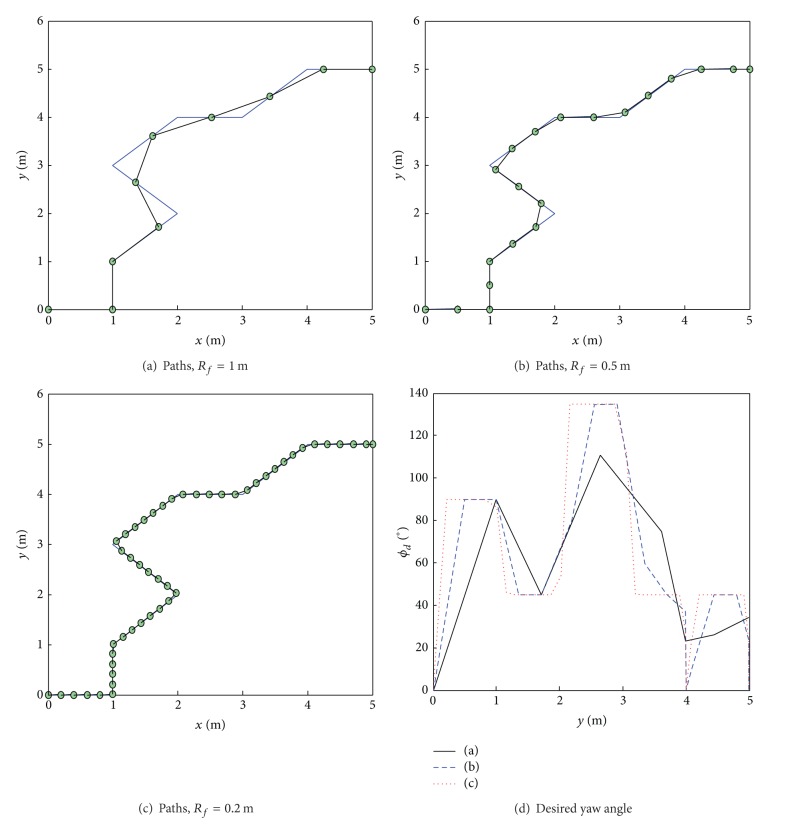
Influence of *R*_*f*_ on reference path and desired yaw angle.

The look-ahead distance, *R*_*f*_, has an obvious influence on the reference path and the desired yaw angle, *ϕ*_*d*_, as shown in Figure 8. A smaller *R*_*f*_ can generate a reference path, the precision of which is greater than that of the planned path. Increasing *R*_*f*_ decreases the precision of the reference path, but the angle *ϕ*_*d*_ varies much less, meaning that the reference path is smoother. The value of *R*_*f*_ should be chosen taking both the exploration requirements and the dimension of the rover into account. In this study, when controlling the El-Dorado II rover, *R*_*f*_ is set to be 0.5 m, a dimension comparable to that of the rover. This dimension can both ensure path-following precision and decrease the steering angle of the rover.

**Figure 9:**
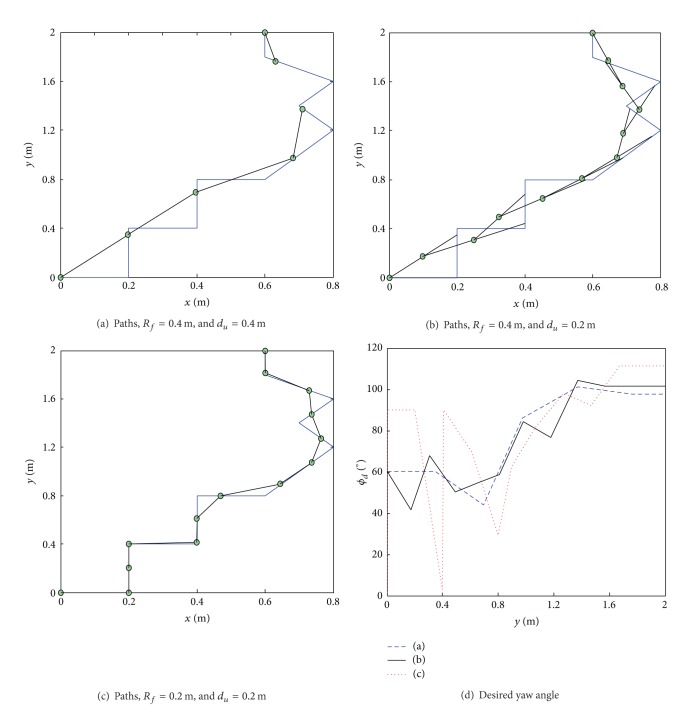
Influence of *d*_*u*_ on reference path and desired yaw angle.

For the DEM to show the small dimensional obstacles or craters that the rover has to overcome, the resolution of the DEM should be comparable to the dimension of the wheels. However, *R*_*f*_ is much larger, compared to the dimensions of the rover's body, in order to smooth the reference path. Because of this difference, the reference path cannot reflect the effective information of the planned path in DEM. For example, in Figure 9(a), the resolution of the DEM is 0.2 m, while *R*_*f*_ is 0.4 m so that the error between the reference path and the planned path is large. In order to solve the problem, a path updating distance, *d*_*u*_, is introduced, the value of which is equal to or smaller than the resolution of the DEM. As soon as the rover moves over a distance *d*_*u*_, the reference path is updated using a look-ahead circle with a radius of *R*_*f*_ so that it can find its next goal point. As shown in Figure 9, the combination of a larger *R*_*f*_ and a smaller *d*_*u*_ has its advantages; the information in the DEM is reflected very well and the steering frequency that is required is decreased. Moreover, a smaller *d*_*u*_ is helpful for finding the deviation caused by the rover's slip and skid and for decreasing the path-following error.

## 4. Strategy of Following the Reference Path

For the rover to follow the reference path and to compensate for the lateral skid, path-following strategy should be studied.

**Figure 10:**
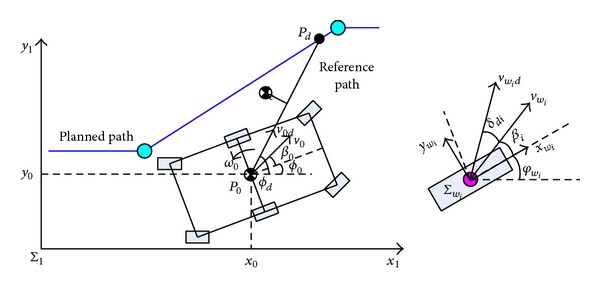
Illustration of path-following control.

Figure 10 shows the path-following control diagram. When the goal point *P*_*d*_ is determined, the reference path is the line *P*_0_ *P*_*d*_. The rover will move from point *P*_0_ = {*x*_0_, *y*_0_} to point *P*_*d*_ = {*x*_*d*_, *y*_*d*_}. *ϕ*_*d*_, the desired yaw angle of the rover, is the included angle between line *P*_0_ *P*_*d*_ and the *x*_*I*_-axis.

When following the reference path, (1) the yaw angle of the rover, *ϕ*_0_, should be close to the desired yaw angle, *ϕ*_*d*_, (2) the velocity of the rover's body should be along its longitudinal direction in order to decrease the lateral skid, and (3) the distance from the rover's center *P*_0_ to the reference path, denoted by *l*_*e*_, should be as small as possible. Let *ϕ*_*e*_ = *ϕ*_*d*_ − *ϕ*_0_ denote the error in the yaw angle. The control objective of path following is *ϕ*_*e*_ → 0, *β*_0_ → 0, and *l*_*e*_ → 0. However, this control objective cannot be directly realized. The variables that can be controlled directly are (1) the angular velocity of the wheels, *ω*_*i*_, and (2) the steering angle of the wheels, *δ*_*i*_.

In order to realize the control objective by coordinating the angular velocities and steering angles of the wheels, the relationship between them should be analyzed. On deformable rough terrain, the motion in the vertical direction, that is, the motion along the *z*-axis, is passively determined by the terrain. The results of studies of path following for a WMR moving on flat, hard terrain therefore provide a basic understanding of this relationship. The path-following objective is realized by controlling the linear velocity *v*_0*d*_ and angular velocity *ω*_0*d*_ of the robots' body, and these velocities are in turn to be controlled by the angular velocities and steering angles of the wheels.

The differences between the control of WMRs on flat, hard terrain and on deformable rough terrain involve the longitudinal slip and lateral skid of wheels and the nonholonomic kinematics models. All the factors except for the longitudinal slip are well considered in this study. The longitudinal slip is characterized by a variable named slip ratio: *s* = (*rω*_*i*_ − *v*_*i*_)/*rω*_*i*_. The slip ratios of the wheels should be coordinated in order to save energy. Since this is not the key issue addressed by this study, the angular velocities of the wheels can be set to constant values. The slip ratios of the different wheels are not equal on rough terrain, and the linear velocity of the rover's body, *v*_0*d*_, is determined mainly by the angular velocity *ω*_*i*_ and the slip ratios.

The remaining problem is how to coordinate the steering angles of the wheels in order to realize the path-following objective. The proportional-integral-derivative (PID) control algorithm is used to generate the angular velocity of the rover's body:

(23) ω0d=kfpϕe+kfdϕ˙e+kfi∫ϕedt+klple|v0d|+kldl˙e|v0d|+kli∫ledt|v0d|+kβpβ0+kβdβ˙0+kbi∫β0dt.

The angular velocity of the rover's body should follow the yaw angle and compensate for lateral skid. Such an angular velocity is realized by the steering motion of the wheels. The forward wheels and the rear wheels can play different roles by using different PID parameters. For example, the forward wheels compensate mainly for the skid and the rear wheels mainly follow the yaw angle. Variables *ω*_0*df*_ and *ω*_0*dr*_ are used to denote the angular velocity of the rover's body that is generated by the forward wheels and the rear wheels. Reference [19] used this concept to control a rover so that it followed a straight line on slopes.

Given the desired velocity of the rover's body, *v*_0*d*_, one can calculate the desired velocity in the inertia coordinate system: ${\dot{x}}_{0d}=v_{0d}\cos\phi_{d}$ and ${\dot{y}}_{0d}=v_{0d}\sin\phi_{d}$. Thus the state variables of the rover's body are

(24) $$\begin{array}{c} {\left[\begin{array}{ccc} {\dot{x}}_{0d} & {\dot{y}}_{0d} & \omega_{0d} \end{array}\right]}^{T}={\left[\begin{array}{ccc} v_{0d}\cos\phi_{d} & v_{0d}\sin\phi_{d} & \omega_{0df}\left(\omega_{0dr}\right) \end{array}\right]}^{T}. \end{array}$$

The state variables of the *i*th wheel in the inertia coordinate system are

(25) $$\begin{array}{c} \left[\begin{array}{c} {\dot{x}}_{w_{i}d}\\ {\dot{y}}_{w_{i}d} \end{array}\right]=\left[\begin{array}{c} v_{0d}\cos\phi_{d}\\ v_{0d}\sin\phi_{d} \end{array}\right]+\left[\begin{array}{ccc} 0 & f_{1} & -f_{2}\\ -f_{1} & 0 & f_{3} \end{array}\right]\left[\begin{array}{c} \dot{\psi }\\ \dot{\theta }\\ \omega_{0d} \end{array}\right]+{\dot{q}}_{r}\left[\begin{array}{c} f_{4}\\ f_{5} \end{array}\right]. \end{array}$$

The angular velocity of the suspension joints, ${\dot{q}}_{r}$, can be measured using angle sensors, such as encoders. The angular velocity of the roll and pitch angle can be measured using the on-board IMU sensors. They can also be neglected for the sake of simplicity, since their values are small:

(26) $$\begin{array}{c} \left[\begin{array}{c} {\dot{x}}_{w_{i}d}\\ {\dot{y}}_{w_{i}d} \end{array}\right]=\left[\begin{array}{c} v_{0d}\cos\phi_{d}\\ v_{0d}\sin\phi_{d} \end{array}\right]+\omega_{0d}\left[\begin{array}{c} -f_{2}\\ f_{3} \end{array}\right]+{\dot{q}}_{r}\left[\begin{array}{c} f_{4}\\ f_{5} \end{array}\right]. \end{array}$$

Substituting the above desired wheel velocity into (22), one obtains the velocity components of the *i*th wheel: [x˙widwiy˙widwi]T. According to the nonholonomic kinematics model of the wheels and Figure 10, we deduce the desired steering angle of the *i*th wheel:

(27) δdi=arctan⁡(y˙widwix˙widwi)−βi.

**Figure 11:**
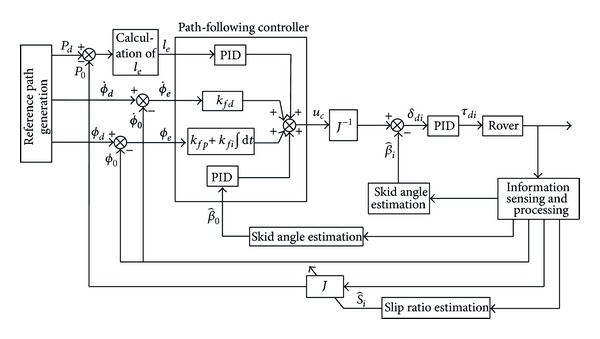
Wheel's steering control strategy for path following.

In order to maneuver the wheels to achieve the steering angle, *δ*_*di*_, a PID controller is used to calculate the torque, *τ*_*di*_, of the steering motors. The path-following strategy is illustrated in Figure 11. The necessary data for control, including the orientation of the rover's body and the position and velocity of the joints, can be measured and processed by sensors. The skid angle and slip ratio of the wheels can be estimated using the methods presented in [23, 24]. The position of the rover's body is calculated by kinematics analysis using the measurement data.

## 5. Simulation Verification

The El-Dorado II rover and the parameters of Toyoura sand were applied in the simulation, and the DEM of rough terrain was generated using MATLAB [20].

The rear wheels were controlled to follow the yaw angle; the PID parameters in (23) were *k*_*fpr*_ = 2, *k*_*fir*_ = 0.1, and *k*_*fdr*_ = 0.5; the others were zero. The forward wheels were controlled to follow the yaw angle and compensate for the lateral skid; the PID parameters in (23) were *k*_*fpf*_ = 0.4, *k*_*fif*_ = 0.02, *k*_*fdf*_ = 0.1, *k*_*βpf*_ = 0.5, *k*_*βif*_ = 0, and *k*_*βdf*_ = 0.1; the others were zero. The lateral skid distance *l*_*e*_ was compensated using a small reference path updating distance *d*_*u*_, the value of which was 0.2 m. The forward angular velocities of all the wheels were 3 rad/s.

**Figure 12:**
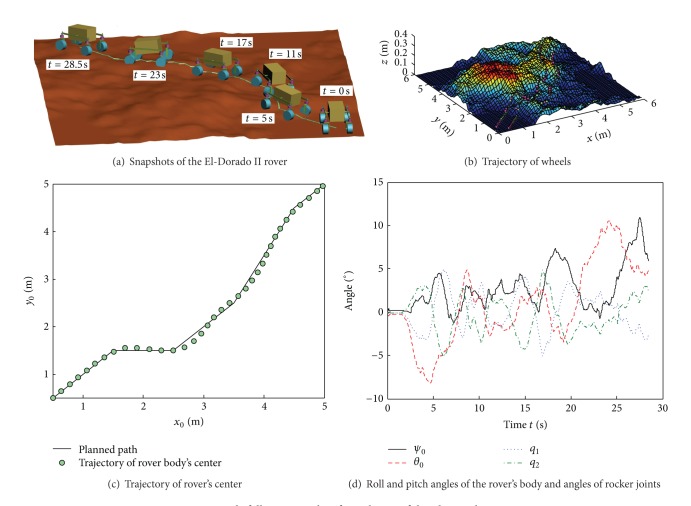
Path-following results of simulation of the El-Dorado II rover.

Figure 12 shows the simulation results of controlling the El-Dorado II rover so that it follows a path in rough deformable terrain.  Figure 12(a) presents the snapshots of the El-Dorado II rover and Figure 12(b) shows the trajectory of the wheels on rough terrain.  Figure 12(c) shows that the center of the rover's body can follow the planned path very well, although there is a slight deviation. If path-following strategy is not applied, the rover would deviate by a large distance from its planned path due to lateral skid, even though the path is a straight line in the horizontal plane. The effectiveness of the path-following algorithm in deformable and rough terrain is verified.  Figure 12(d) shows the roll angle *ψ*_0_ and pitch angle *θ*_0_ of the rover's body and the angles of the rocker joints. When moving on rough terrain, the rocker joints rotate passively in order to keep all the wheels in contact with the terrain and *q*_1_ = −*q*_2_. The rover's body also rolls and pitches to adapt to the rough terrain.

**Figure 13:**
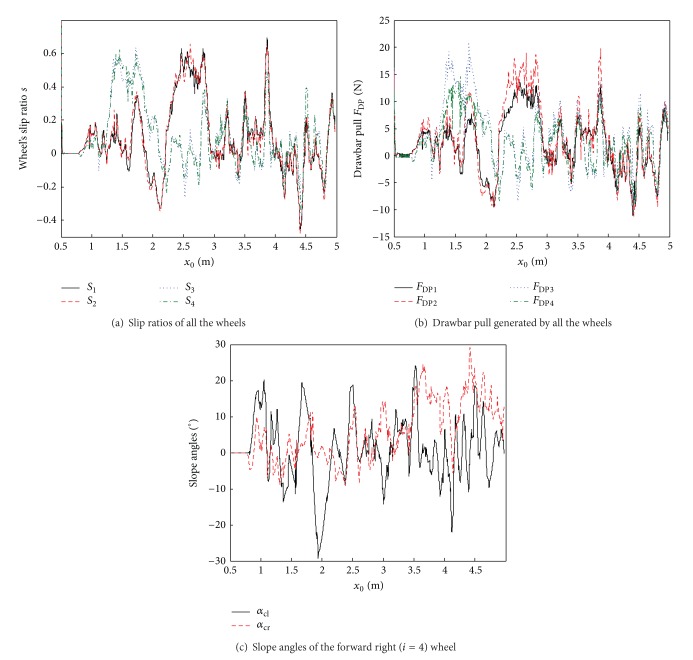
Simulation results for the wheels of El-Dorado II rover.

The curves in Figure 13 denote the simulation results for the wheels of the El-Dorado II rover. The slip ratios and the drawbar pull (the net traction force generated by the wheel) of the wheels are different as they are at different slopes. The varying trend of the drawbar pull is similar to that of the slip ratio [25], which can be explained by terramechanics.  Figure 13(c) shows the slope angles of *α*_cl⁡_ and *α*_cr_ of the forward right (*i* = 4) wheel. The varying trends of the slip ratio and drawbar pull are primarily determined by the angle of *α*_cl⁡_, which is also demonstrated by the curves.

## 6. Conclusions

This paper presents a path-following control method for wheeled planetary exploration robots moving on deformable, rough terrain. The modeling of a WMR on such challenging terrain includes a geometric model of the wheel-terrain contact area and a nonholonomic kinematics model. The coordinate systems of the rover's body and of the wheels are different, and their transformation matrix to the inertia coordinate system can be described using the derived equations presented in this paper. In order to follow the path planned in DEM, the reference path should be updated in real time. By combining a longer look-ahead distance and a shorter path updating distance, the DEM information can be reflected very well and the actual path is smoothed to decrease the steering motion of the rover. The path following of a WMR is primarily achieved by controlling the angular steering velocity of the rover's body, which in turn is realized by coordinating the position of the steering wheels. The path-following strategy for a WMR moving on deformable and rough terrain is designed. Different PID parameters can ensure that the forward and rear wheels play different roles in terms of following the yaw angle and compensating for the lateral skid. The four-wheeled El-Dorado II rover is used in a simulation experiment, and the effectiveness of the path-following strategy in deformable and rough terrain is verified.

## Figures and Tables

**Table 1:** Position coordinates and yaw angles of the planned path.

Number	1	2	3	4	5	6	7	8	9
*x*/*x*_*r*_ (m)	0	1	1	2	1	2	3	4	5
*y*/*y*_*r*_ (m)	0	0	1	2	3	4	4	5	5
*ϕ(°)*	0	π/2	π/4	3π/4	π/4	0	π/4	0	
